# Supplementary figures and images for: Transcription levels and prognostic significance of the NFI family members in human cancers
Source: PeerJ. 2020 Mar 18;8:e8816. doi: 10.7717/peerj.8816 (PMC7085295; doi:10.7717/peerj.8816)

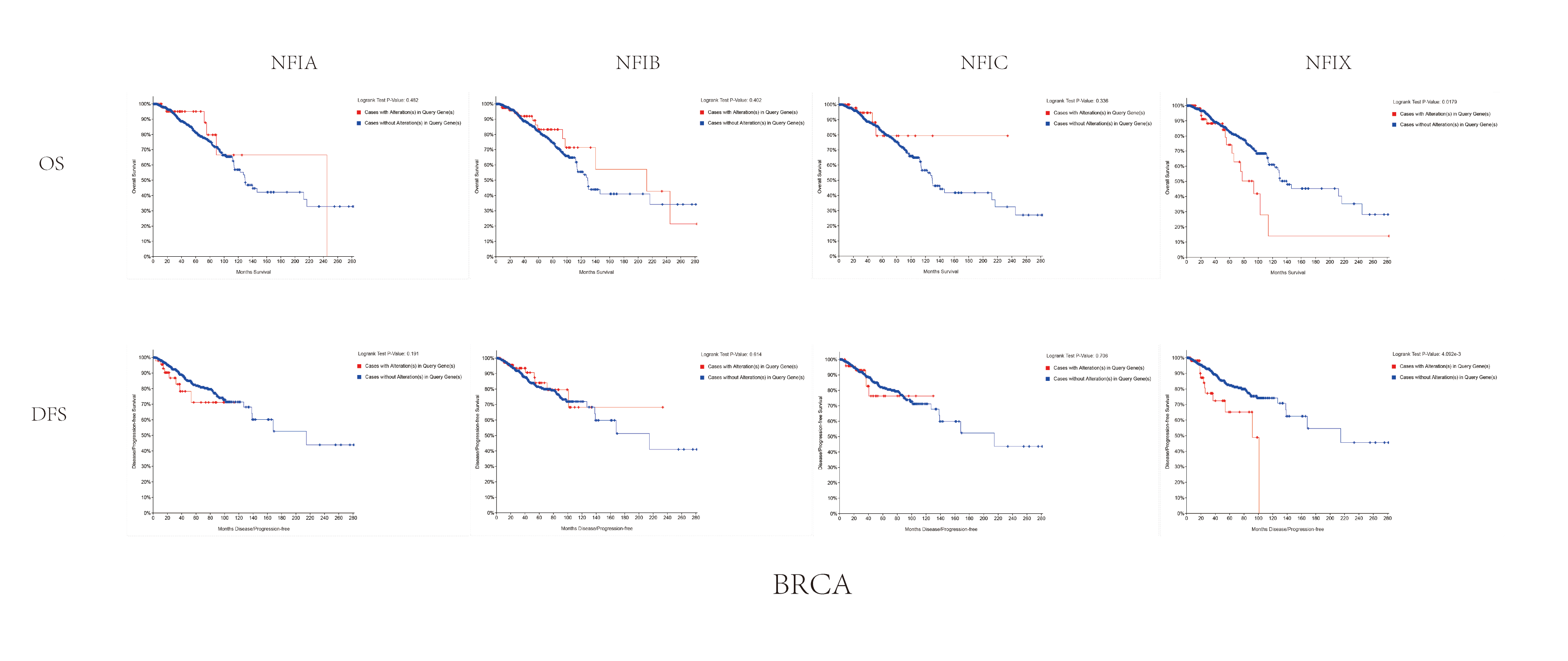

Supplement: Supplemental Information 1 — Notes: Blue color shows cases without NF Is alterations and red color shows cases with NFIs alterations. Abbreviations: BRCA, breast cancer. [file peerj-08-8816-s001.png]

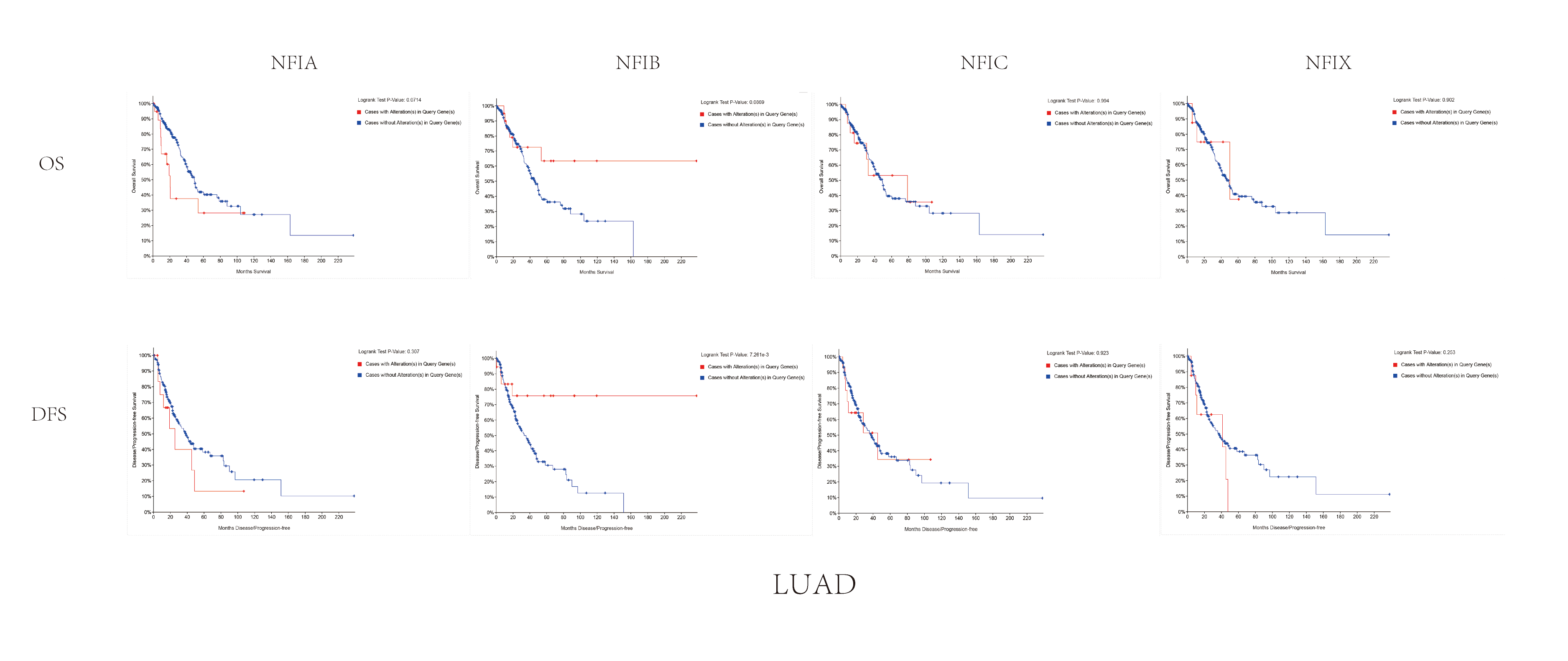

Supplement: Supplemental Information 2 — Notes: Blue color shows cases without NFIs alterations and red color shows cases with NFIs alterations. Abbreviations: LUAD, lung adenocarcinoma. [file peerj-08-8816-s002.png]

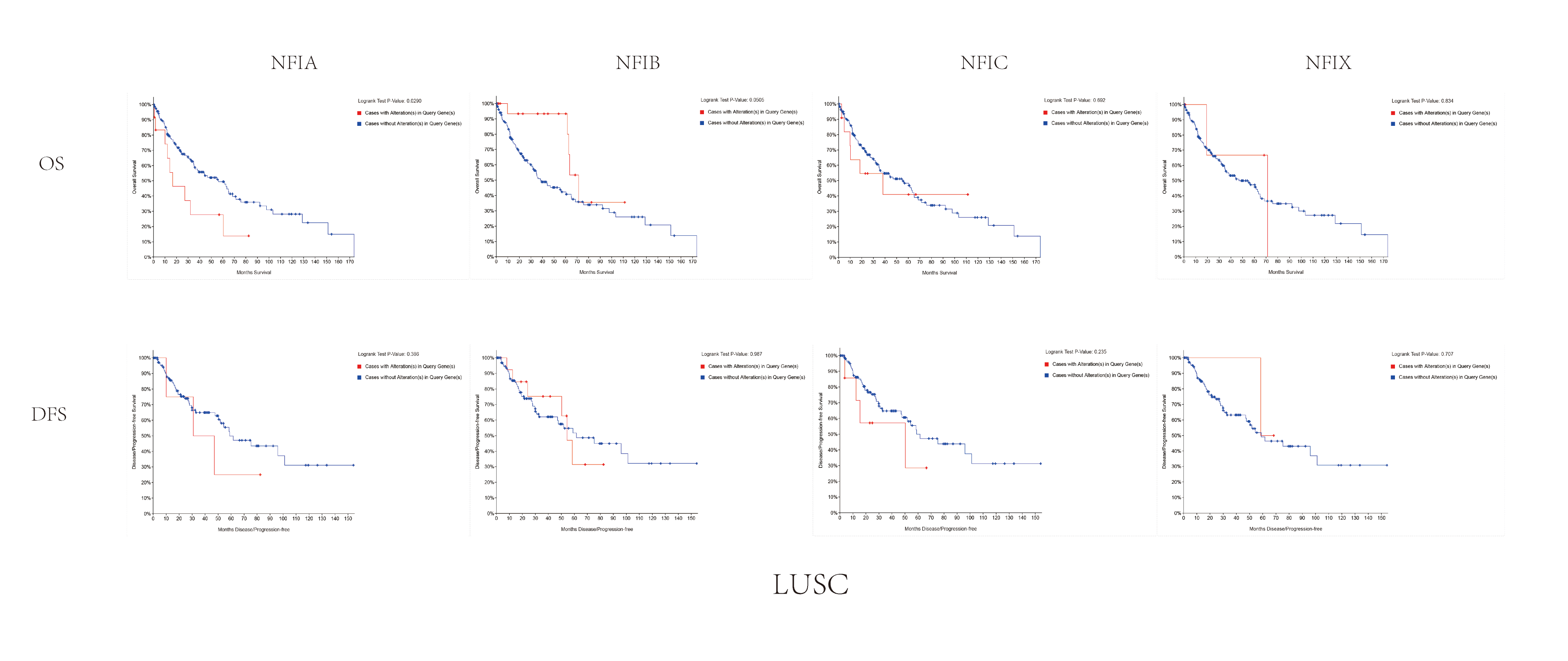

Supplement: Supplemental Information 3 — Notes: Blue color shows cases without NFIs alterations and red color shows cases with NFIs alterations. Abbreviations: LUSC, lung squamous cell carcinoma. [file peerj-08-8816-s003.png]

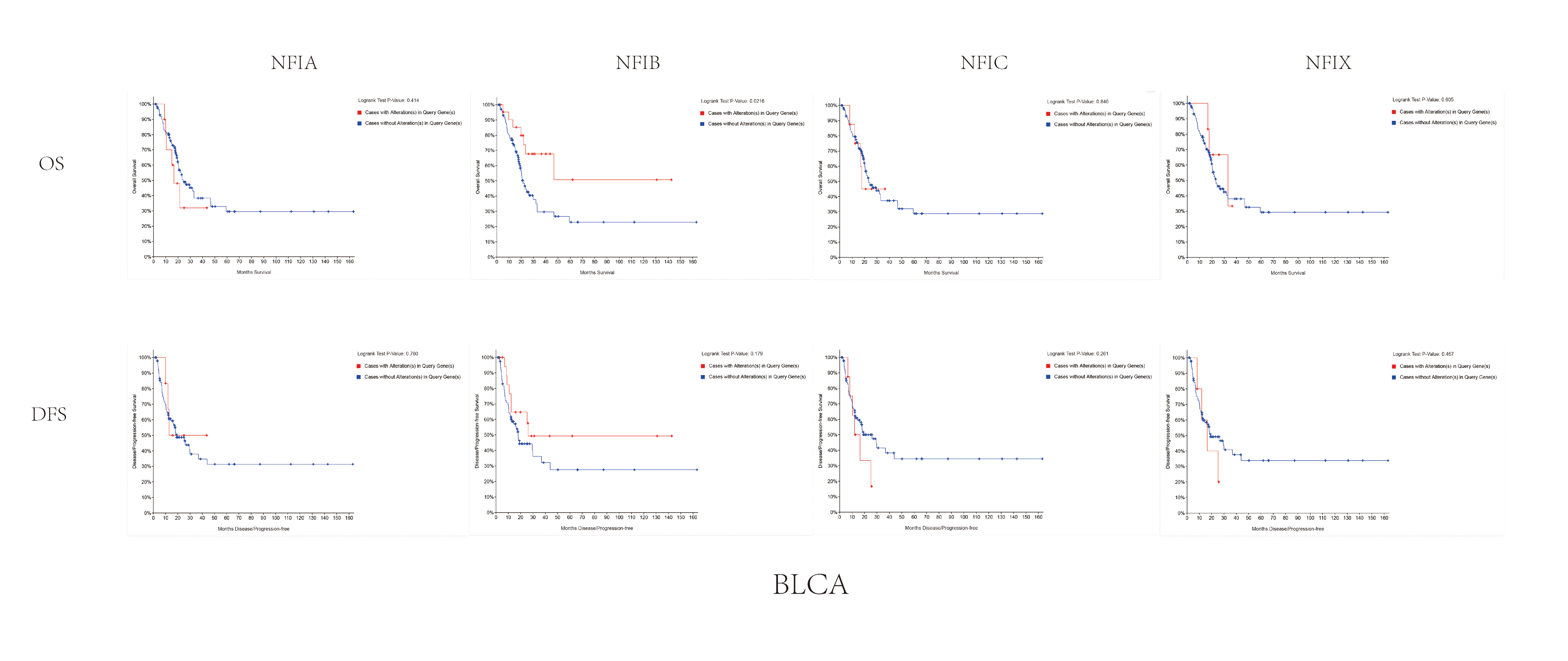

Supplement: Supplemental Information 4 — Notes: Blue color shows cases without NFIs alterations and red color shows cases with NFIs alterations. Abbreviations: BLCA, bladder cancer. [file peerj-08-8816-s004.png]

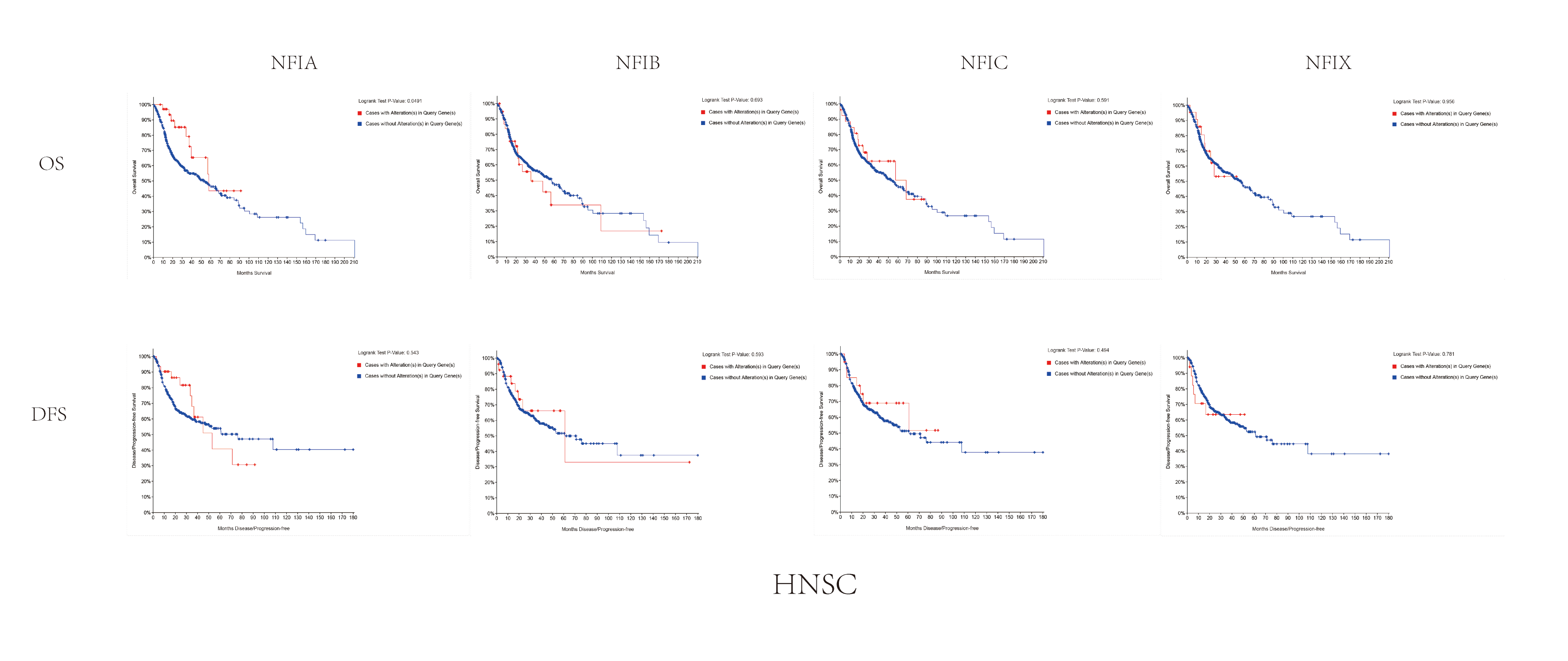

Supplement: Supplemental Information 5 — Notes: Blue color shows cases without NFIs alterations and red color shows cases with NFIs alterations. Abbreviations: HNSC, head and neck cancer. [file peerj-08-8816-s005.png]

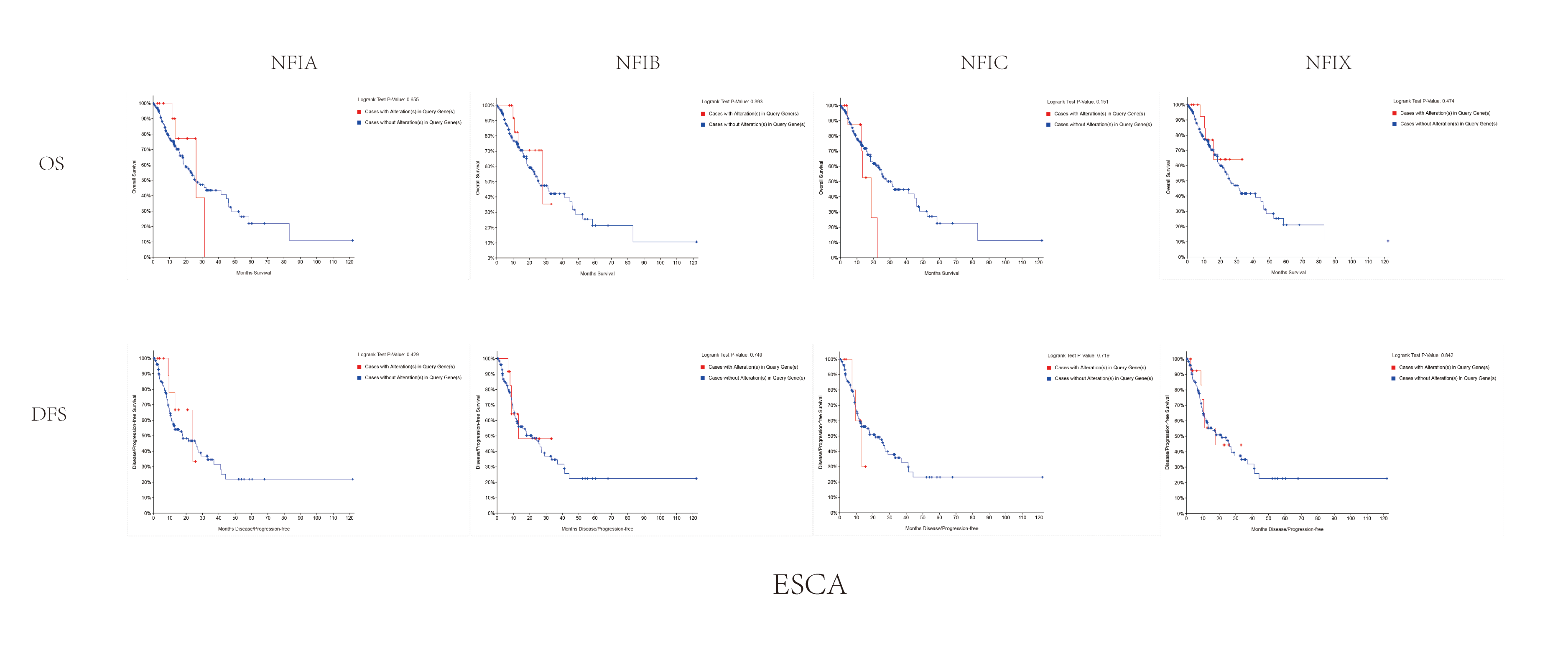

Supplement: Supplemental Information 6 — Notes: Blue color shows cases without NFIs alterations and red color shows cases with NFIs alterations. Abbreviations: ESCA, esophageal cancer. [file peerj-08-8816-s006.png]

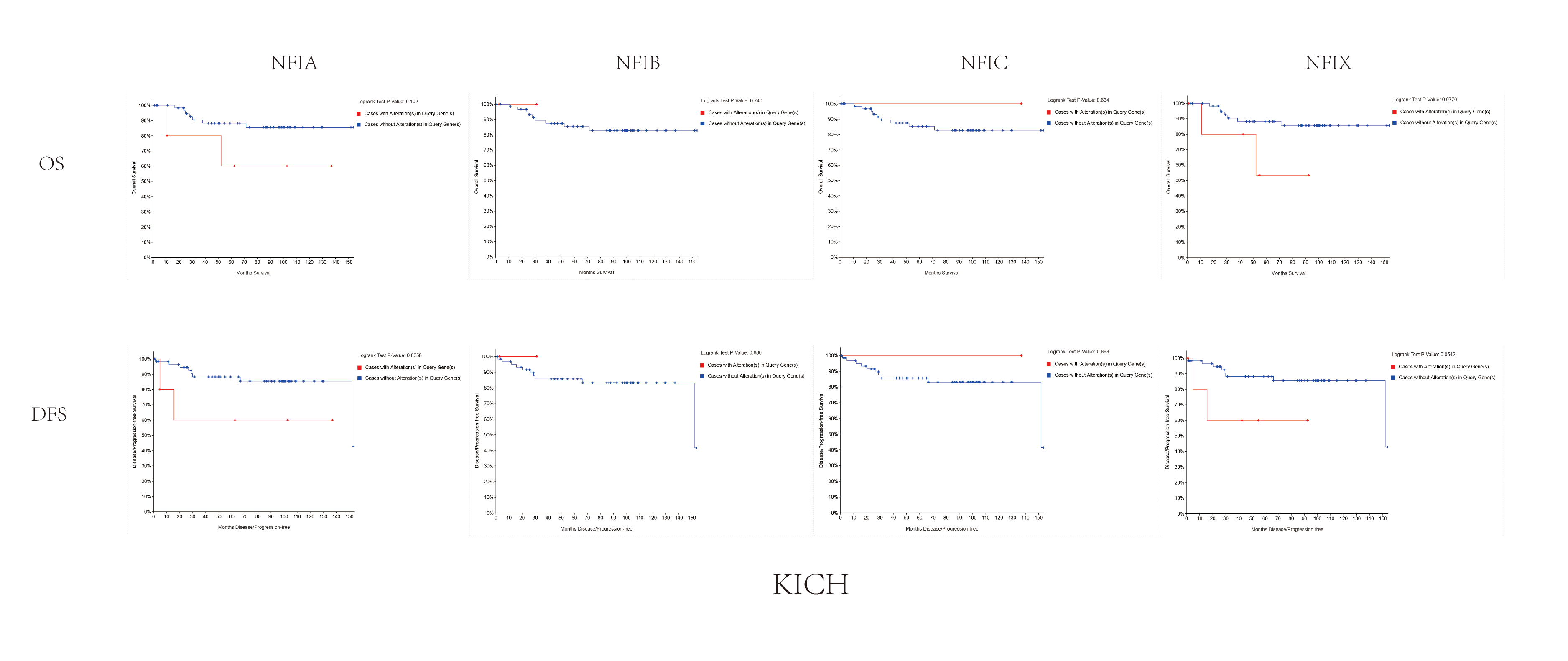

Supplement: Supplemental Information 7 — Notes: Blue color shows cases without NFIs alterations and red color shows cases with NFIs alterations. Abbreviations: KICH, kidney chromophobe. [file peerj-08-8816-s007.png]

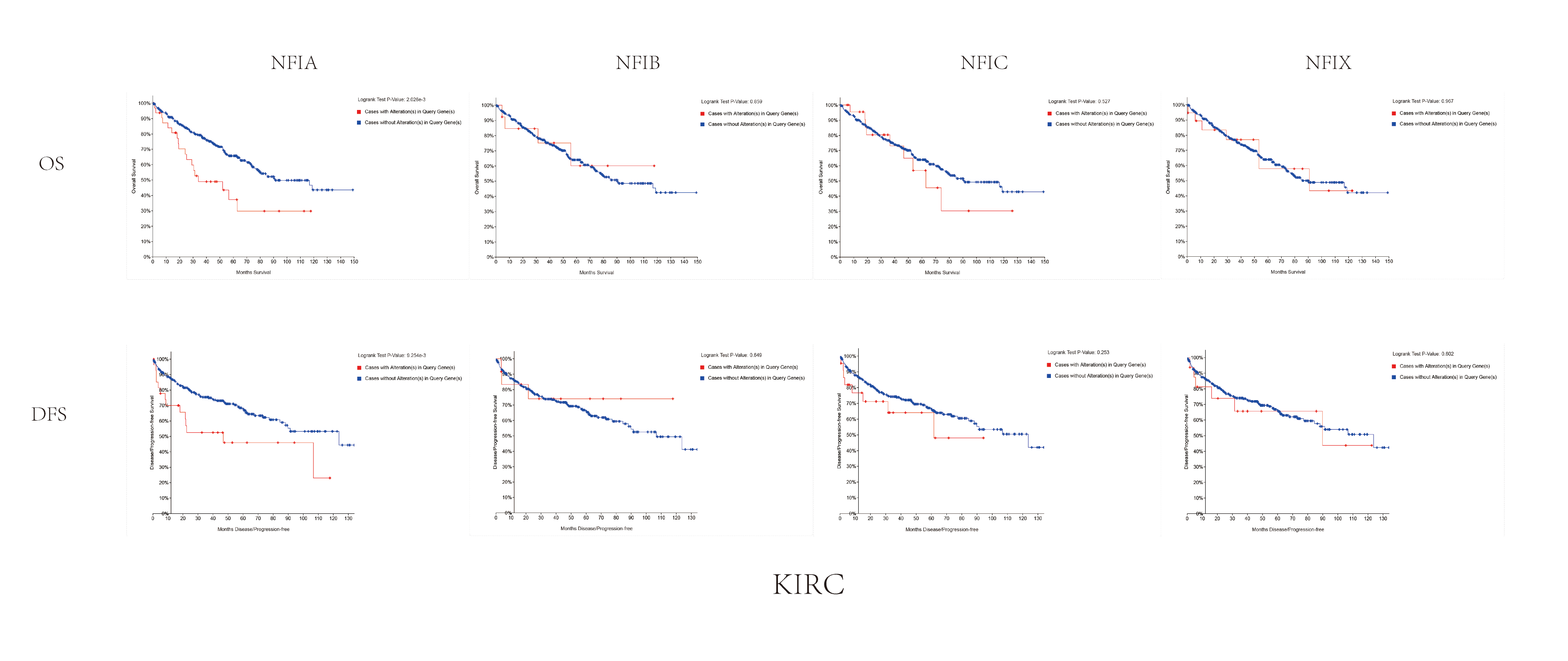

Supplement: Supplemental Information 8 — Notes: Blue color shows cases without NFIs alterations and red color shows cases with NFIs alterations. Abbreviations: KIRC, kidney clear cell carcinoma. [file peerj-08-8816-s008.png]

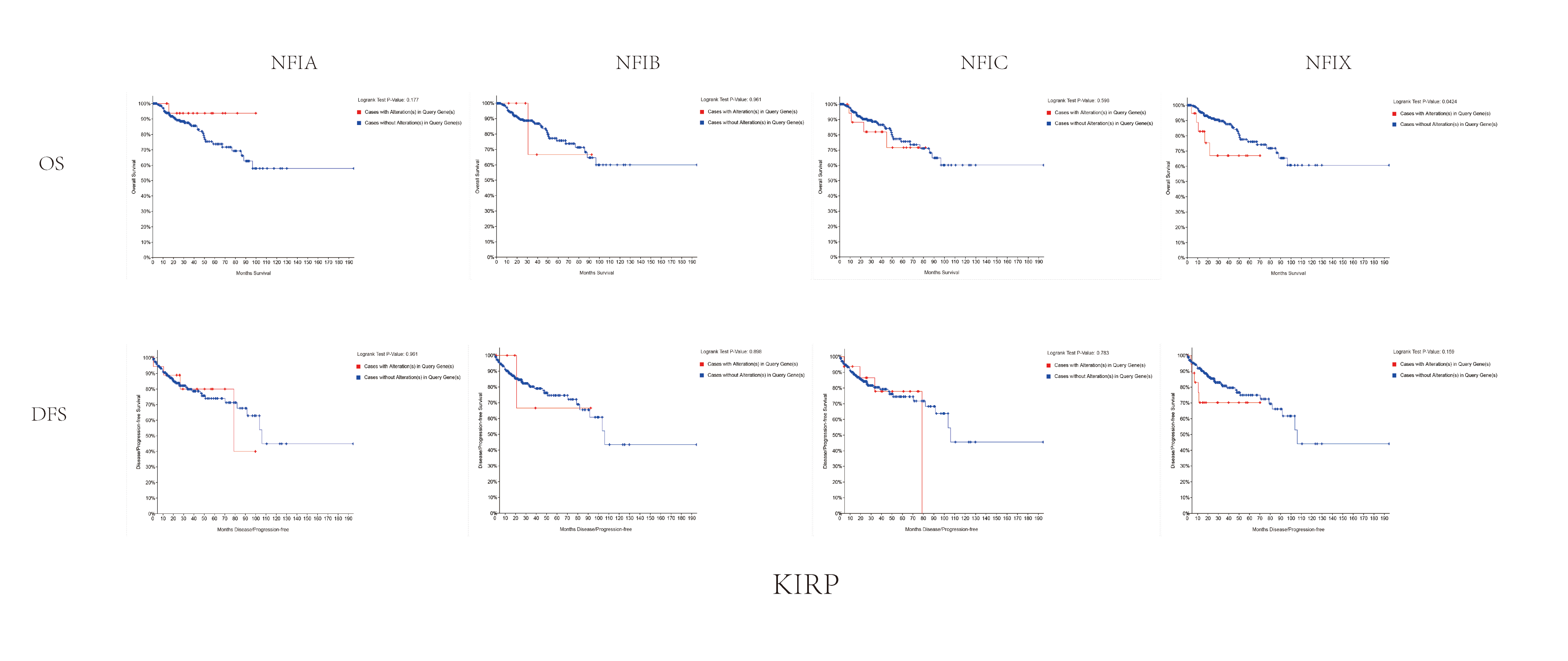

Supplement: Supplemental Information 9 — Notes: Blue color shows cases without NFIs alterations and red color shows cases with NFIs alterations. Abbreviations: KIRP, kidney papillary cell carcinoma. [file peerj-08-8816-s009.png]

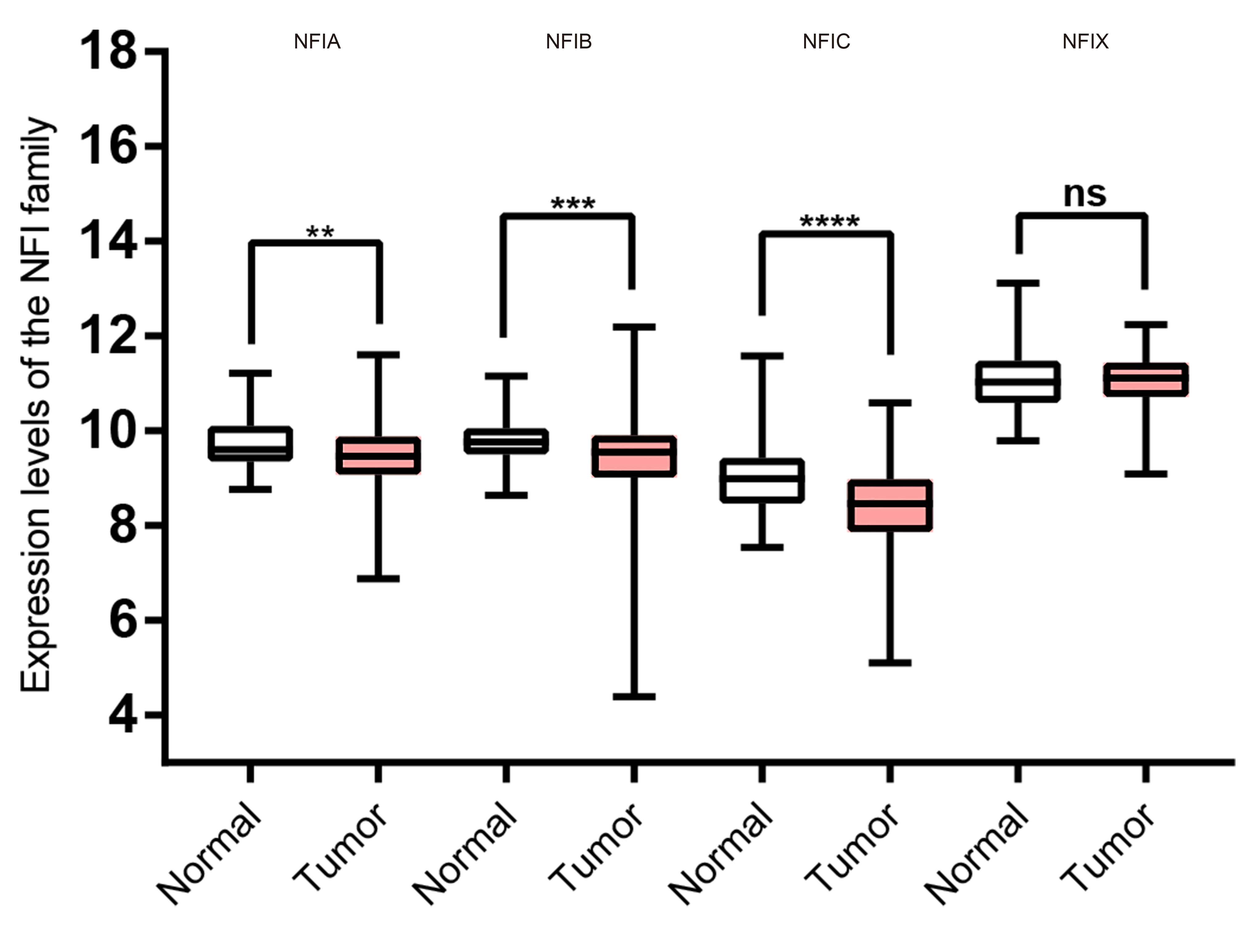

Supplement: Supplemental Information 10 — Box-whisker plots show the differences in transcript levels of the NFI family members between normal and tumors samples. The median value is represented by the middle line in the boxes. Statistical differences were examined by two tailed student’s t-test. ****p < 0.0001, ***p < 0.001, **p < 0.01, no-non significant. Abbreviations: NFI, Nuclear factor I; TCGA, The Cancer Genome Atlas. [file peerj-08-8816-s010.png]

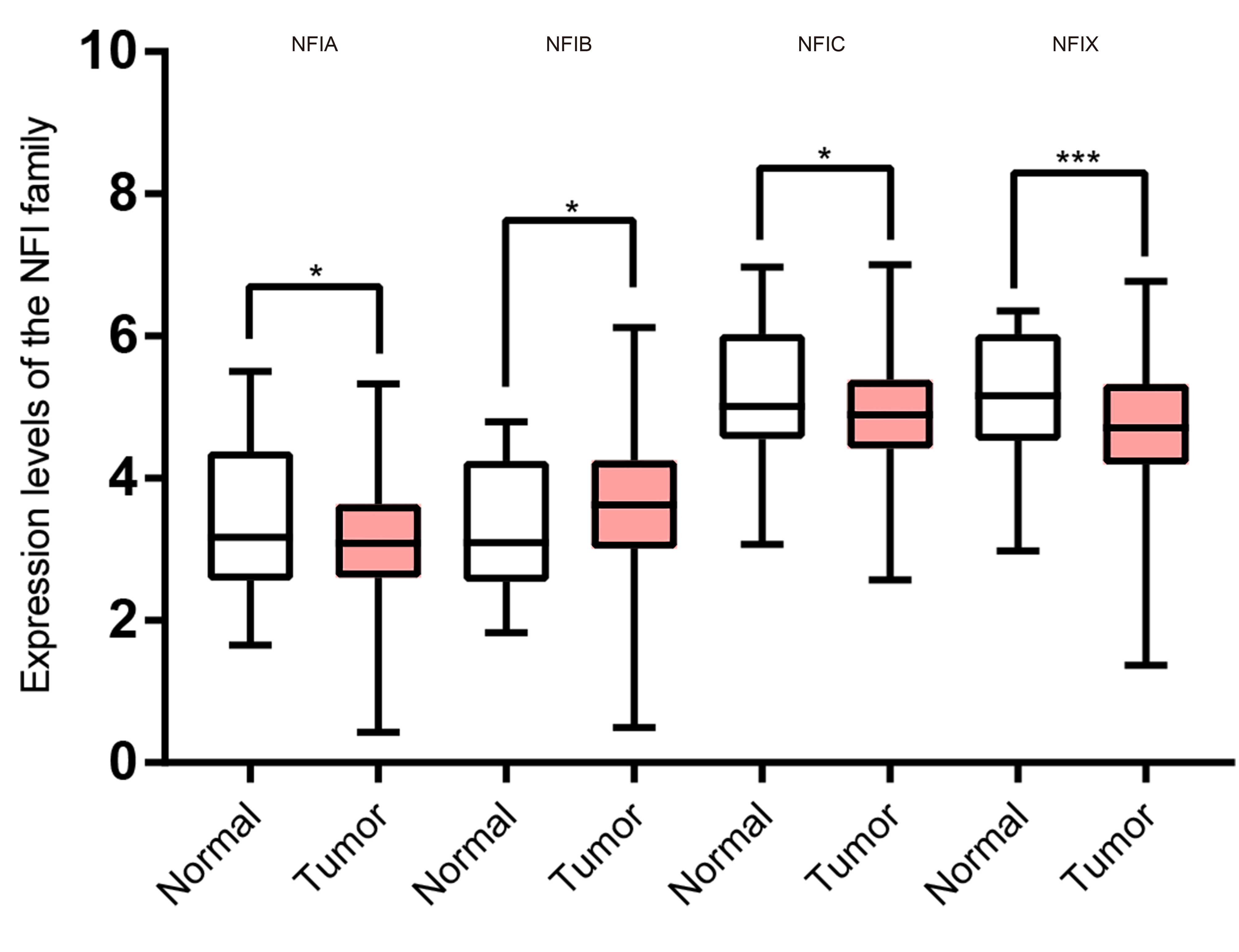

Supplement: Supplemental Information 11 — Box-whisker plots show the differences in transcript levels of the NFI family members between normal and tumors samples. The median value is represented by the middle line in the boxes. Statistical differences were examined by two tailed student’s t-test. ***p < 0.001, * p < 0.05. Abbreviations: NFI, Nuclear factor I; TCGA, The Cancer Genome Atlas. [file peerj-08-8816-s011.png]

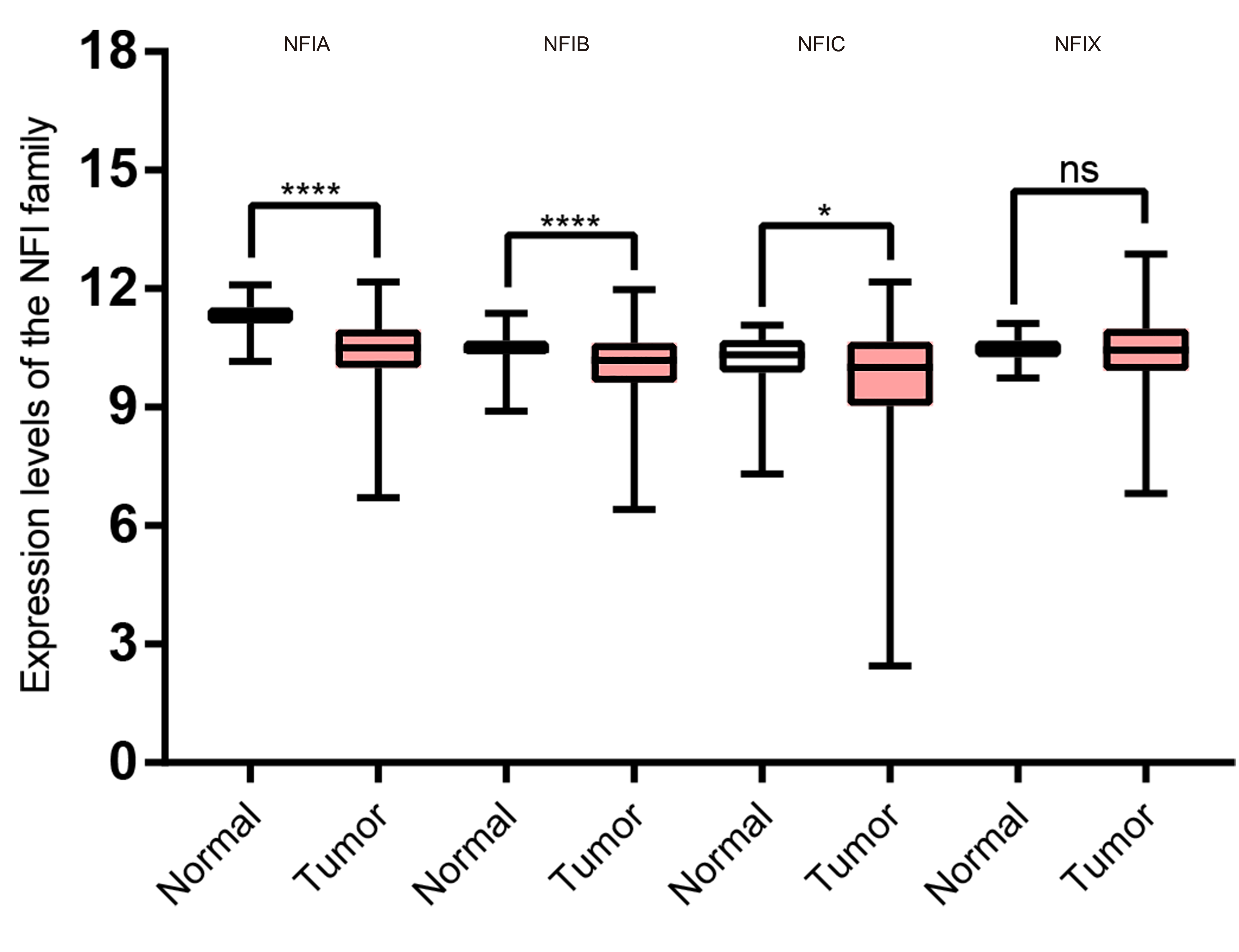

Supplement: Supplemental Information 12 — Box-whisker plots show the differences in transcript levels of the NFI family members between normal and tumors samples. The median value is represented by the middle line in the boxes. Statistical differences were examined by two tailed student’s t-test. ****p < 0.0001, * p < 0.05, ns-non significant. Abbreviations: NFI, Nuclear factor I; TCGA, The Cancer Genome Atlas. [file peerj-08-8816-s012.png]

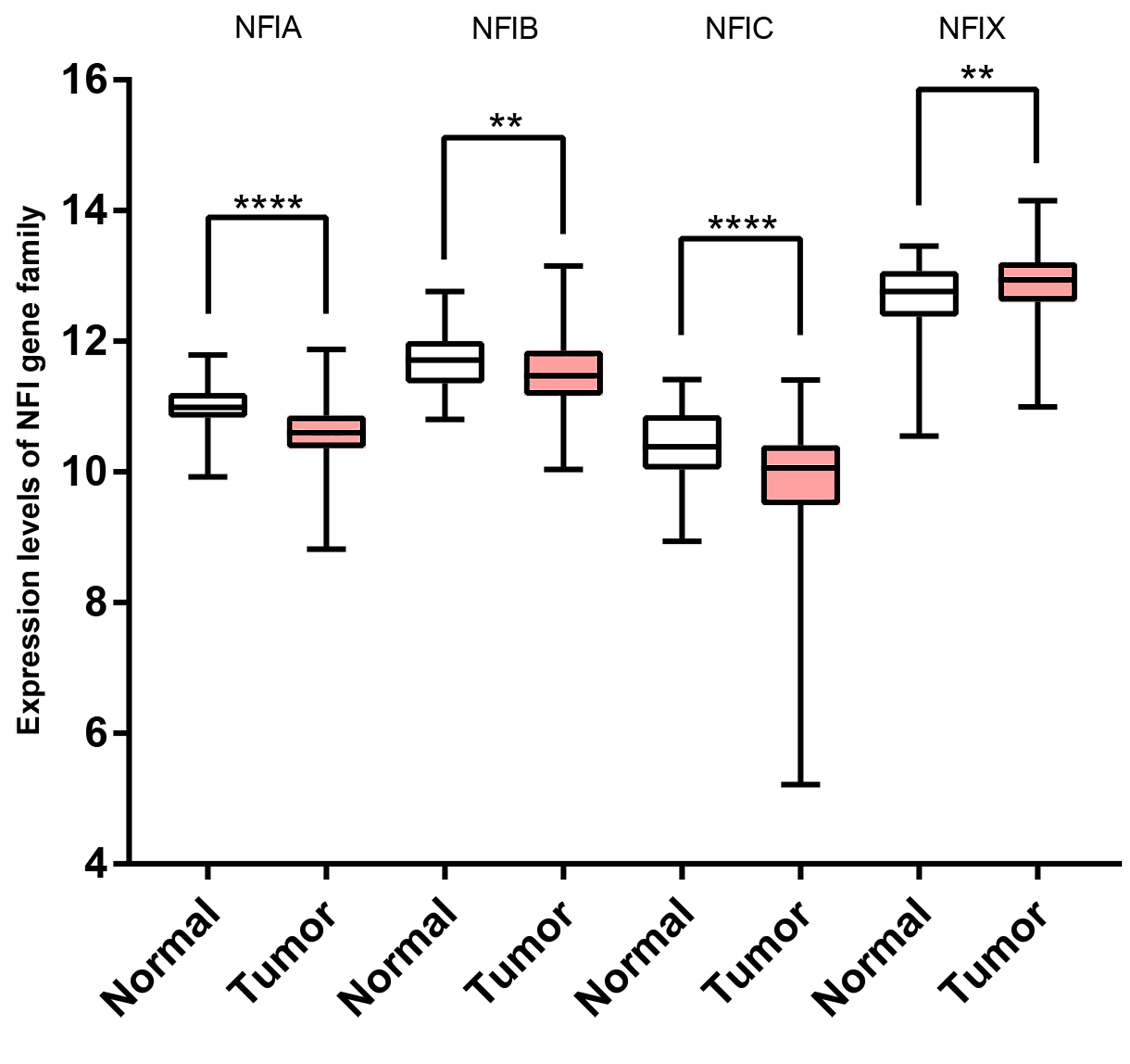

Supplement: Supplemental Information 13 — Notes: Box-whisker plots show the differences in transcript levels of the NFI family members between normal and tumors samples. The median value is represented by the middle line in the boxes. Statistical differences were examined by two tailed student’s t-test. ****p < 0.0001, **p < 0.01. Abbreviations: NFI, Nuclear factor I; TCGA, The Cancer Genome Atlas. [file peerj-08-8816-s013.png]

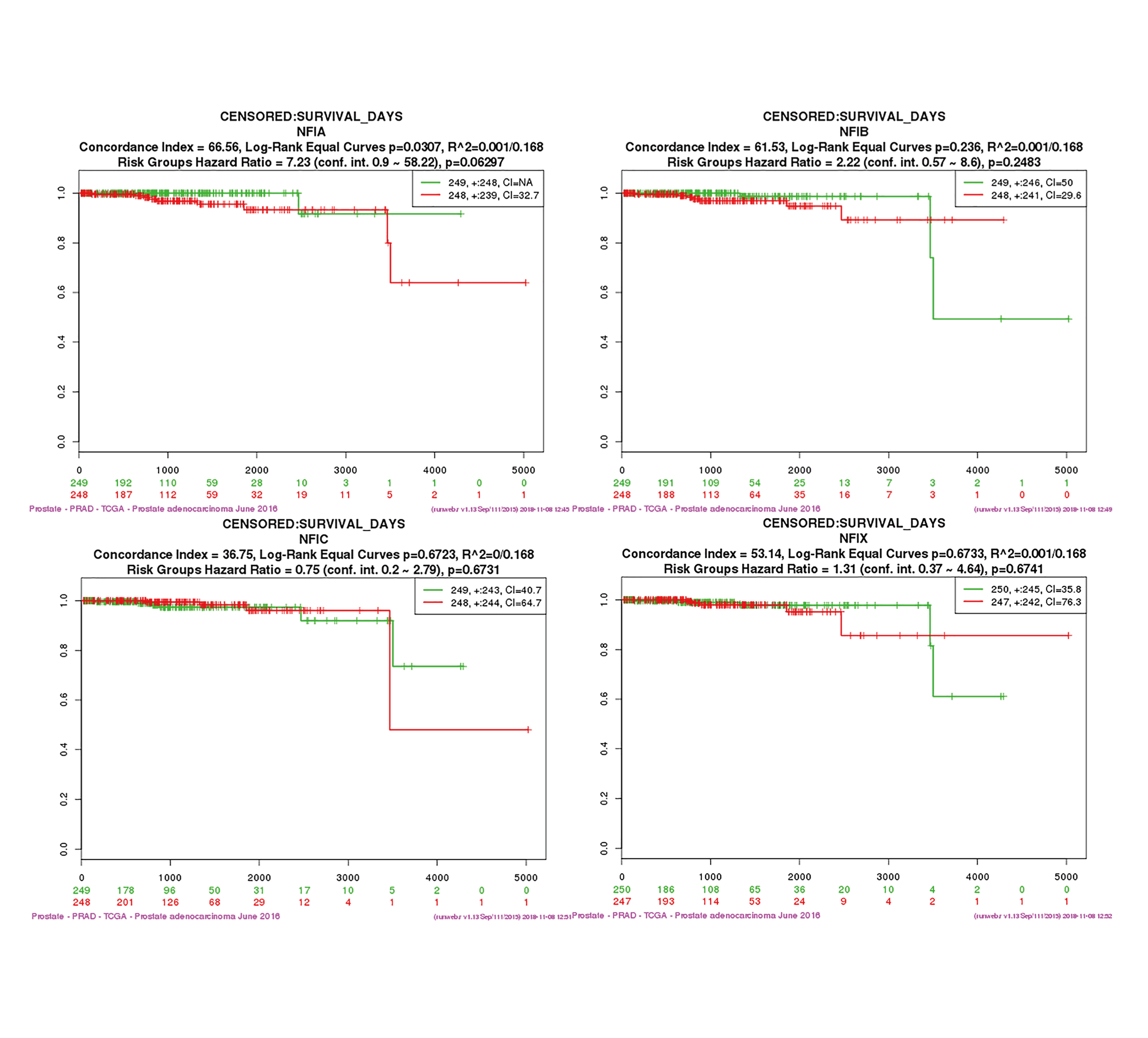

Supplement: Supplemental Information 14 — Green color shows low risk group and red color shows high risk group. Abbreviations: TCGA, The Cancer Genome Atlas; PRAD, Prostate adenocarcinoma. [file peerj-08-8816-s014.png]
