# Supplementary material for: Transcription levels and prognostic significance of the NFI family members in human cancers
Source: PeerJ. 2020 Mar 18;8:e8816. doi: 10.7717/peerj.8816 (PMC7085295; doi:10.7717/peerj.8816)
Supplement: Supplemental Information 15 — S1a. Correlation of NFIs with survival outcomes in breast cancer(basal-like) patients; S1b.Correlation of NFIs with survival outcomes in breast cancer(HER2+) patients; S1c.Correlation of NFIs with survival outcomes in breast cancer(luminal A) patients S1d.Correlation of NFIs with survival outcomes in breast cancer(liminal B) patients [file peerj-08-8816-s015.docx]

**Abbreviations:** HR, hazard ratio; CI, confidence interval; OS, overall survival; RFS, relapse free survival; DMFS, distant metastasis free survival; PPS, post progression survival.
