# Supplementary material for: Transcription levels and prognostic significance of the NFI family members in human cancers
Source: PeerJ. 2020 Mar 18;8:e8816. doi: 10.7717/peerj.8816 (PMC7085295; doi:10.7717/peerj.8816)
Supplement: Supplemental Information 16 — S2a Correlation of NFIs with survival outcomes in lung adenocarcinoma patients S2b Correlation of NFIs with survival outcomes in lung squamous cell carcinoma patients [file peerj-08-8816-s016.docx]

**Abbreviations:** HR, hazard ratio; CI, confidence interval; OS, overall survival; FP, first progression; PPS, post progression survival.
