# Supplementary material for: Transcription levels and prognostic significance of the NFI family members in human cancers
Source: PeerJ. 2020 Mar 18;8:e8816. doi: 10.7717/peerj.8816 (PMC7085295; doi:10.7717/peerj.8816)
Supplement: Supplemental Information 24 [file peerj-08-8816-s024.docx]

******Abbreviations:** HR, hazard ratio; CI, confidence interval; OS, overall survival; RFS, relapse free survival; PFS, progression free survival; DSS, disease specific survival.
