# Supplementary material for: Transcription levels and prognostic significance of the NFI family members in human cancers
Source: PeerJ. 2020 Mar 18;8:e8816. doi: 10.7717/peerj.8816 (PMC7085295; doi:10.7717/peerj.8816)
Supplement: Supplementary file 7 [file peerj-08-8816-s017.docx]

Table S3 Datasets of the NFI family in cervical cancer (ONCOMINE database)

Gene	Dataset	Normal (cases)	Tumor (cases)	Fold change	
NFIB	Zhai Cervix	Cervix Squamous Epithelium(10)	High Grade Cervical Squamous(7)	-2.207	
	Scotto Cervix 2	Cervix Squamous Epithelium(24)	Cervical Squamous Cell Carcinoma(37)	-2.377	
NFIC	Biewenga Cervix	Cervix Uteri(5)	Cervical Squamous Cell Carcinoma(40)	-2.384	
