# Supplementary material for: Transcription levels and prognostic significance of the NFI family members in human cancers
Source: PeerJ. 2020 Mar 18;8:e8816. doi: 10.7717/peerj.8816 (PMC7085295; doi:10.7717/peerj.8816)
Supplement: Supplementary file 8 [file peerj-08-8816-s019.docx]

Table S5	Datasets of the NFI family in colorectal cancer (ONCOMINE database)				
**Gene**	**Dataset**	**Normal（cases)**	**Tumor（cases)**	**Fold change**	
NFIA	TCGA Colorectal	Normal(colon(19) rectum(3))	Rectosigmoid Adenocarcioma(3)	-3.563	
			Cecum Adenocarcinoma(22)	-3.529	
	Kaiser Colon	Normal(colon(5))	Rectal Mucinous Adenocarcinoma(4)	2.757	
			Cecum Adenocarcinoma(17)	2.919	
NFIB	Skrzypczak Colorectal 2	Normal(colon(10))	Colon Adenoma	2.204	
	TCGA Colorectal	Normal(colon(19) rectum(3))	Cecum Adenocarcinoma(22)	-2.395	
