# Supplementary material for: Transcription levels and prognostic significance of the NFI family members in human cancers
Source: PeerJ. 2020 Mar 18;8:e8816. doi: 10.7717/peerj.8816 (PMC7085295; doi:10.7717/peerj.8816)
Supplement: Supplementary file 9 [file peerj-08-8816-s021.docx]

Table S7	Datasets of the NFI family in gastric cancer (ONCOMINE database)					
**Gene**	**Dataset**	**Normal（cases)**	**Tumor（cases)**	**Fold change**	**t-test**	
NFIB	DErrico Gastric	Gastric Mucosa(31)	Gastric Mixed Adenocarcinoma(4)	3.247	5.495	
NFIX	DErrico Gastric	Gastric Mucosa(31)	Gastric Mixed Adenocarcinoma(4)	-3.061	-5.885	
