# Supplementary material for: Transcription levels and prognostic significance of the NFI family members in human cancers
Source: PeerJ. 2020 Mar 18;8:e8816. doi: 10.7717/peerj.8816 (PMC7085295; doi:10.7717/peerj.8816)
Supplement: Supplementary file 10 [file peerj-08-8816-s023.docx]

Table S9 Datasets of the NFI family in liver hepatocellular carcinoma (ONCOMINE database)

Gene	Dataset	Normal（cases）	Tumor（cases）	Fold change	t-test
NFIA	Wurmbach Liver	Normal(Liver(10))	Hepatocellular Carcinoma(35)	-3.017	-5.093
			Cirrhosis(13)	-3.178	-3.921
			Liver Cell Dysplasia(17)	-2.505	-3.149
