# Supplementary material for: Transcription levels and prognostic significance of the NFI family members in human cancers
Source: PeerJ. 2020 Mar 18;8:e8816. doi: 10.7717/peerj.8816 (PMC7085295; doi:10.7717/peerj.8816)
Supplement: Supplementary file 11 [file peerj-08-8816-s025.docx]

Table S11 Datasets of the NFI family in prostate cancer (ONCOMINE database)

Gene	Dataset	Normal（cases）	Tumor（cases）	Fold change	t-test
NFIB	Wallace Prostate	Prostate Gland(20)	Prostate Adenocarcinoma(69)	3.572	4.409
	Tomlins Prostate	Prostate Gland(23)	Prostate Carcinoma(29)	-2.276	-4.407
NFIC	Luo Prostate	Prostate Gland(15)	Prostate Carcinoma(15)	-6.752	-2.764
NFIX	Tomlins Prostate	Prostate Gland(21)	Benign Prostate Hyperplasia(4)	3.362	3.794
	Varambally Prostate	Prostate Gland(6)	Prostate Carcinoma(7)	-3.092	-3.053
